# What motivates new, established and long-term users of herbal medicine: is there more than push and pull?

**DOI:** 10.1186/s12906-019-2584-7

**Published:** 2019-07-10

**Authors:** Alexandra N. Welz, Agnes Emberger-Klein, Klaus Menrad

**Affiliations:** TUM Campus Straubing for Biotechnology and Sustainability, Weihenstephan-Triesdorf University of Applied Sciences, Petersgasse 18, 94315 Straubing, Germany

**Keywords:** Herbal medicine, CAM, Reasons for use, Online survey, Factor analysis

## Abstract

**Background:**

The use of herbal medicine (HM) has become an essential form of treatment and it is more and more common around the world. Little is known about the reasons that drive people to initially use HM or to maintain their behaviour, and whether the so-called “push and pull factors” known in the context of decision making for complementary and alternative medicine, also play a role for HM use. Here, our goal was to provide answers to these open questions and to analyse the reasons that motivate new, established and long-term HM consumers in detail.

**Methods:**

Thirteen reasons for HM usage, which were previously identified within a qualitative approach, were analysed quantitatively in a nationwide online survey in Germany. Data of 2,192 German HM users from the general population were grouped into new, established and long-term users. We performed a factor analysis in order to identify factors underlying the set of reasons.

**Results:**

We discovered a reliable factor associated with longstanding family traditions and cultural importance of HM in Germany. This finding shows that the reasons for HM use require a three-factor structure going beyond the well-known push and pull factors that explain the use of complementary and alternative medicine. In using the identified factors for further calculations, we were able to reveal important group differences and test how the factor scores perform as predictors for the new, established and long-term choice of HM. Our results showed that a high score on the push factor is associated more with initial HM usage, while long-term HM usage is impacted more by high scores on the pull and traditional factors.

**Conclusions:**

Our exploratory survey and analysis of the reasons that underlie HM usage aimed at providing a better understanding of the decision for this treatment form. The findings of our work deliver insights for medical practitioners and health-care providers, including the role of family traditions for HM usage and the finding that new HM users are driven to use this treatment form in part because of negative aspects they associate with conventional medicine.

## Background

Usage of complementary and alternative medicines (CAM) has been increasing worldwide during the past decades [1]. Herbal medicine (HM) as a treatment form belongs to the class of “biologically based CAM treatments” [2] and has often been found to be among the most popular and strongest growing forms of CAM therapies. In the European Union, HM prevalence rates were reported to be high in general as shown in the review paper by Eardley et al. [3] who demonstrated prevalence rates for HM use of around 50% for various countries. Tindle et al. [4] compared data from 1997 to 2002 and found that HM has the greatest relative increase of all CAM treatment forms in the US, with prevalence rates soaring from 12.1 to 18.6%. And a nationwide study in Germany has reported growing numbers of HM users with prevalence rates increasing from 52% in 1970 to 70% in 2010 [5]. There is no doubt that HM has a high and growing importance in today’s health care, and is relevant for people in managing their health and illnesses. This has also been emphasised by the WHO, which classified HM as an essential component of primary healthcare [6].

So far, only few studies have considered the decision making process [7–9], which includes the motivation of people for choosing HM initially or in a repeated way. What is known so far about why people use HM? We will start to address this question by first providing an overview of previous research on the reasons for CAM use, which has been studied far more than the use of its subgroup HM. However, large variations of different CAM therapies make it difficult to transfer research results from one treatment form to another. Keeping this difficulty in mind, the following five reasons for CAM were examined in the seminal NHIS study in USA: (1) thought it would be interesting to try; (2) thought herbs combined with conventional treatment would help; (3) conventional treatment would not help; (4) conventional medical provider suggested herbs; and (5) conventional treatments were too expensive. Results of previous scientific studies show that these reasons form a solid basis for researching reasons for CAM usage [10–12]. However, the results of a recent qualitative study specifically for HM in Germany showed that there appear to be more than just these five reasons motivating people to choose it as a treatment [13]. Moreover, even if one could transfer reasons for CAM to HM use, one would still be limited by the fact that health-care systems in the US differ widely from those in the EU and that, as mentioned above, many different treatment forms are summarised as “CAM”. Considering all of these issues advocates for a complex structure of multiple reasons, which is also supported by the review paper of Eardley et al., who analysed 18 studies on CAM conducted in the EU, and who reported that all of these studies were using different sets of reasons for CAM usage.

In search for a more general structure underlying the reasons for CAM use, it was found that they could be categorised into “push” and “pull” factors. Push factors are negative aspects associated with CM, e.g., ineffectiveness [14], dissatisfaction with the relationship to the conventional physician [15, 16] or side-effects of conventional medicinal products [17] “pushing” people from CM to CAM. Pull factors, on the other hand, are positive aspects associated with CAM, such as holistic beliefs or the expectation of fewer side effects, “pulling” people more into the CAM direction [18]. It is still an open question whether the push [19, 20] or the pull factors [18–25] have a greater influence on the overall decision for CAM use, and whether they can also determine the duration of CAM usage, i.e., comparing new to long-term users [14, 26, 27].

In contrast to the case of CAM, the reasons for the use of HM have hardly been analysed so far. Thus, it is currently unknown if comparable categories of push and pull factors even exist for HM, nor what influence they have on the initial or long-term use of HM. Moreover, in the context of reasons for HM use, we mostly found studies that had examined this issue for specific population subgroups. For example, Damery et al. [28] explored reasons for why cancer patients were using HM and found they did so in order to “reduce symptoms associated with cancer” or to “address associated conditions”. Clearly, while these were important reasons for this specific group, such results cannot be transferred to the general population.

In order to get a general picture on the situation in Germany, we investigated the reasons for usage of HM in the general German population in this study by going beyond a purely descriptive statistical analysis. We chose the general population (beyond specific subgroups) as the target group for this study because previous research showed that the aims for HM use go beyond treating illnesses and include preventing illnesses and promoting health [29, 30], i.e. people who also do not suffer from specific conditions use HM and must have reasons for doing so. Thus, based on results of a pre-study, in which we have identified 13 different reasons with focus group discussions for HM usage on the basis of a qualitative approach, we first of all undertook a nationwide online survey in the general population of Germany. With this approach, we were able to identify which of these reasons for HM usage found the largest degree of agreement among people who used HM in the previous year. We then performed the critical step of testing whether the known CAM push and pull factors could be verified as reasons for HM usage, and by factor analysis whether additional factors existed in this context. This approach allowed us to examine the differences in the importance of each identified factor between new, established and long-term users of HM, and last but not least, for analysing how these factors perform as predictor for the duration of using HM.

With these findings, we provided important insights into why people decide to use herbs as medicinal treatment in Germany, which is essential e.g. for health-care providers and governmental bodies and can therefore support the optimisation of patient care. Differentiating between new, established and long term users of HM contributes to a better understanding of the different drivers behind the initial use of HM and its long-term application, or in other words, what motivates people to initiate HM usage and what convinces them to maintain this behaviour. Our findings contribute to the ongoing discussion of which factor has the relative greatest influence on the overall, initial and repeated decision making for the important CAM subgroup of HM.

## Method

### Study design and data collection method

We conducted a nationwide online survey concerning HM usage in the German 18+ population via an online panel provided by a market-research institute. The survey was conducted from January to February 2018. Before starting the questionnaire, participants gave their informed consent, and after finishing the questionnaire, they received a monetary incentive. The Ethics Commission of the Faculty of Medicine, Technical University of Munich, approved the study on January 8, 2018.

### Questionnaire design, sections and items

The questionnaire was constructed entirely by the authors, and was based on established measurement scales as well as on results of a qualitative pre-study. It consists of several sections, and it is noted that a section about HM usage pattern, established psychological measurement scales, and a discrete choice experiment are not part of this paper but will be discussed elsewhere. For answering the research questions of this work that were mentioned above, the following items were used in the calculations:

People answering the screening item as used in the important US American NHIS surveys by the National Center for Health Statistics “*Have you used herbal medicine in the last 12 months for your own health or treatment*” with ‘*yes*’ (1) instead of ‘*no*’ (2) received further questions about the duration of HM use *(“How long have you been taking herbal medicine for your own health or illness”*), with response options including *‘for the past year or less’* (1), *‘for longer than one year’* (2), and *‘since adolescence’* (3). This defined our three user groups “new”, “established” and “long-term” HM users. Furthermore, the questionnaire asked about a series of sociodemographic and health related variables, whereby results regarding the following ones will be reported in this publication: gender, age, years of education, size of household, occupation, chronic disease, and self-perceived health-status. Moreover, participants had to rate their level of agreement for 13 reasons of HM usage derived in the focus group discussions on a five point Likert-type scale, from ‘*strongly disagree*’ (1), ‘*disagree*’ (2), ‘*neither agree nor disagree*’ (3), ‘*agree*’ (4) to ‘*strongly agree*’ (5). We note that HM was defined in our questionnaire as all plant-derived products including their natural form, as well as pills derived from extracts.

### Participants

The total sample of 2,906 people represented the general German population with regard to gender, age, federal state, size of household and residence. Two thousand one hundred ninety-two people had agreed that they had used HM in the previous 12 months. The respective distribution of sociodemographic and health related variables of the respondents can be found in Table 1.

**Table 1:** User characteristics of the overall HM users in the previous year and the three user subgroups and results of Chi-Square tests for a subgroup comparison

Variables	HM user in previous year (*n* = 2192) %	New user *(n = 166)* %	Established user *(n = 1482)* %	Long-term user *(n = 544)* %	χ^2^ (df); *p* value
Total N	100	7.6	67.6	24.8	
Gender					
Male	44.5	54.2	45.2	39.7	χ^2^(2) = 11.708; *p* = .003
Female	55.5	45.8	54.8	60.3	
Age group (years)					
18–29	17.6	24.1	12.6	29.2	χ^2^(8) = 95.228; *p* ≤ .0005
30–39	13.0	16.9	12.6	13.1	
40–49	17.0	10.8	17.9	16.4	
50–59	19.1	13.3	20.9	16.2	
≥ 60	33.3	34.9	36.0	25.2	
Years of Education					
< 12 Years	47.4	50.0	52.7	32.4	χ^2^(2) = 66.534; *p* ≤ .0005
≥ 12 Years	52.6	50.0	47.3	67.7	
Size of household					
1	23.0	21.7	23.4	22.2	χ^2^(6) = 12.265; *p* = .056, n.s.
2	42.8	37.6	42.8	41.4	
3	16.7	12.7	17.8	14.9	
≥ 4	17.5	18.1	16.0	5.3	
Occupation					
Employed	66.0	56.6	66.5	67.6	χ^2^(2) = 7.301; *p* = .026
Unemployed	34.0	43.4	33.5	32.4	
Self-perceived health status					
Very good, good	63.0	57.8	61.4	68.9	χ^2^(2) = 11.740; *p* = .003
Fair, poor, very poor	37.0	42.2	38.6	31.1	
Chronic disease					
Yes	56.6	57.8	57.2	54.4	χ^2^(2) = 1.394; *p* = .498, n.s.
No	43.4	42.2	42.8	45.6	

n.s. denotes *p* > 0.05

### Statistical analysis

Descriptive statistics was used for analysing sample characteristics and the degree of agreement to the set of reasons. We decided for the use of an exploratory factor analysis approach instead of a confirmatory one, because the number of factors and dispersion of items was not clear a priori [31]: while we expected a minimum of two relevant factors, i.e. the push and pull factor known in the context of CAM, we did not know whether there will be further ones, hence the use of an exploratory approach. The number of derived factors was obtained using those factors with eigenvalues larger than 1 in combination with Cattell’s scree plot. In line with recommendations by Russel [32] and Bühner [32], we used the principle-axis factor extraction technique with promax rotation for determining whether one or more factors underlie the pool of items on why people use HM. We chose the promax rotation, because we assumed correlations between the factors. Factor mean values were computed by averaging the relevant item scores and were used for further calculations. For group comparisons, we used Welch ANOVA instead of the traditional analysis of variance, because our data violate the assumption of homogeneity of variances and the Welch’s test is a more robust statistical method in this case [33]. For post-hoc comparisons between the groups, we followed the recommendations given by Field [34] and decided for the Games-Howell post hoc test. For identifying the influence of the derived factors in discriminating the three user groups, a multinomial logistic regression analysis was performed. All significance tests were conducted on the *p* < 0.05 level. We used the statistics software package SPSS for Windows, release 23, for all of the calculations.

## Results

In this section, we will first show the results of sociodemographic and health-related information of the participants who have used HM for the past 12 months and the three user groups, i.e., the new, established and long-term HM users (vide infra). Secondly, results are provided concerning the user ratings on the reasons for HM usage and thirdly the results of factor analysis. The last part of this section presents the results of a user group comparison using the identified factor scores in a logistic regression analysis, showing the influence of the analysed factors for being a member of one of the three user groups.

### Duration of herbal medicine use and user characteristics

Of the 2,192 people who answered that they used HM in the last 12 months, 7.6% are what we defined as new HM users, i.e., they used HM initially within the previous 12 months; 67.7% were classified as what we defined as established HM user, i.e., practicing HM usage for more than 1 year; and 24.8% were long-term HM users, in our definition using herbs since adolescence.

Table 1 shows the distribution of sociodemographic variables, the self-perceived health status and whether participants had a chronic disease or not in the defined user groups and the entire sample. Compared to the other two user groups, the new HM users are more likely male (54.2% vs. 45.2 and 39.7%), unemployed (43.4% vs. 33.5 and 32.4%), and rated their health status as fair, poor or very poor (42.2% vs. 38.6 and 31.1%) more frequently. Compared to the long-term user, the new user had an education lasting less than 12 years (50.0% vs. 32.4%) more frequently. As shown in Table 1, significant group differences were also found for the variable “age”, but not for the variables “size of household” and “having a chronic disease”. It should be noted that people who were 60 years or older resembled the largest fraction of participants (33.3%), because our sample reflected the general German population. This needs to be taken into account when comparing the absolute values of frequencies reported in Table 1.

### Reasons for using herbal medicine

Among the overall HM users in the previous year, it was found that reasons highlighting the natural character of HM (‘*I take HM because they are more natural than chemically synthesised medicinal products.*’), as well as positive previous experiences with this kind of treatment (‘*I take HM because I had positive experiences with herbal medicinal products in the past.*’), were the most agreed ones. The reasons with the lowest level of rated agreement for the overall sample and therefore the least important aspects for deciding on HM usage were ‘*I take HM because chemically synthesised medicinal products did not show treatment succes.*’, and ‘*I take HM because I was dissatisfied with the conventional medical practitioner.*’

Comparing the mean values separately for the three user groups, it is apparent that long-term HM users have an overall higher level of agreement than the two other user groups, as manifested in higher mean values on almost all items. Performing a Welch’s ANOVA showed significant statistical differences in the mean values between the three user groups for all the reasons, except for the item ‘*I was dissatisfied with the conventional medical practitioner.*’ All results can be found in Table 2.

**Table 2:** Mean values of the agreement of the reasons for HM use and results of Welch’s ANOVA comparing the subgroups

	Mean (SD)	Subgroup Means (SD)			
I Take HM because...	Total sample *(n = 2192)*	New user *(n = 166)*	Established user *(n = 1482)*	Long-term user *(n = 544)*	Welch’s ANOVA (F ratio)
Chemically synthesised medicinal products have too many side effects.	3.5 (1.1)	3.2 (1.2)	3.5 (1.1)	3.6 (1.2)	5.46**
Chemically synthesised medicinal products have too strong side effects.	3.4 (1.1)	3.1 (1.2)	3.4 (1.1)	3.4 (1.2)	4.77**
Chemically synthesised medicinal products did not show treatment success.	2.5 (1.0)	2.5 (1.0)	2.5 (1.0)	2.4 (1.0)	3.17*
I was dissatisfied with the conventional medical practitioner.	2.6 (1.1)	2.5 (1.2)	2.5 (1.1)	2.6 (1.2)	.94, n.s.
In the past, I had positive experiences with herbal medicinal products.	4.0 (0.9)	3.5 (1.1)	4.0 (0.8)	4.2 (0.8)	43.32***
They have had a positive impact on my health.	3.9 (0.9)	3.4 (1.9)	3.8 (0.8)	4.1 (0.8)	33.16***
They are healthier than chemically synthesised medicinal products.	3.9 (1.0)	3.7 (1.1)	3.9 (1.0)	4.0 (1.0)	4.72**
They are more natural than chemically synthesised medicinal products.	4.1 (0.9)	3.8 (1.0)	4.0 (0.9)	4.2 (0.9)	13.62***
They have a higher tolerability than chemically synthesised medicinal products.	3.8 (1.0)	3.6 (1.0)	3.8 (1.0)	3.9 (1.0)	5.27**
They have fewer side effects than chemically synthesised medicinal products.	3.9 (1.0)	3.7 (1.1)	4.0 (0.9)	4.0 (1.0)	4.08*
I trust HM more than chemically synthesised medicinal products.	3.2 (1.1)	3.0 (1.1)	3.2 (1.1)	3.3 (1.2)	5.31**
In my family we have always used HM.	3.3 (1.1)	2.8 (1.2)	3.1 (1.1)	3.8 (1.0)	89.66***
I am very familiar with herbal medicinal products since my childhood.	3.3 (1.2)	2.6 (1.2)	3.0 (1.2)	4.2 (0.8)	381.25***

Reasons measured on a 5-point Likert Scale: 1 = Strongly disagree, 2 = Disagree, 3 = Neither agree nor disagree, 4 = Agree, 5 = Strongly agree; MEAN = mean value; SD = standard deviation. *p ≤ 0.05; **p ≤ 0.01; ***p ≤ 0.001; n.s. denotes *p* > 0.05

### Identifying factors underlying the reasons for the use of herbal medicine

Concerning the results of the factor analysis, the calculated Kaiser-Meyer-Olkin measure of sampling adequacy [35] was well-above the recommended 0.6 (KMO = 0.880), and Bartlett’s test of sphericity [36] reached the requested statistical significance (*p* ≤ .0005).

Cattell’s scree test [37] as well as Kaiser’s eigenvalue [38] showed a three factor structure, which together explained 67.1% of the overall variance. Factor 1 was comprised of six items related to reasons highlighting positive aspects of HM (“pull factor”), with factor loadings ranging from .571 to .848. Factor 2 included five items related to reasons combining negative aspects concerning CM (“push factor”), with factor loadings ranging from .433 to .772. The statement ‘*I take HM because I trust HM more than chemically synthesised medicinal products*’ *eo ipso* appears as a reason associated with a pull factor, but according to the results of the performed factor analysis showed the highest factor loading on the push factor. We assume that this could be explained by participants understanding this statement probably as ‘*I take HM because I distrust chemically synthesised medicinal products more than HM*’ rather than what was written in the questionnaire. Due to the probable misunderstanding of this item, and the rather high factor loadings of this item also on the other two factors, we have decided not to include this item in further calculations, e.g. in calculating the factors’ mean value. Factor 3 consisted of two items, both associated with family traditions (“traditional factor”), with factor loadings .752 and .738. All of the results of the factor analysis are presented in Table 3.

**Table 3:** Results of factor loadings using principle axis factor analysis with promax rotation

I take HM because …	Factor		
	1	2	3
	Positive aspects of HM (Pull)	Negative aspects of CM (Push)	Traditional aspect (Tradition)
They are more natural than chemically synthesised medicinal products.	**.848**	−.103	
They have fewer side effects than chemically synthesised medicinal products.	**.798**		−.107
They are healthier than chemically synthesised medicinal products.	**.764**		
They have a higher tolerability than chemically synthesised med. Products.	**.727**		
In the past, I had positive experiences with herbal medicinal products.	**.600**	−.206	.318
They have had a positive impact on my health.	**.571**	−.147	.283
Chemically synthesised medicinal products did not show treatment success.	−.189	**.772**	
I was dissatisfied with the conventional medical practitioner.	−.111	**.771**	
Chemically synthesised medicinal products have too strong side effects.	.413	**.518**	−.116
Chemically synthesised medicinal products have too many side effects.	.450	**.505**	−.127
I trust HM more than chemically synthesised medicinal products.	.256	**.433**	.221
In my family we have always used HM.		.134	**.752**
I am very familiar with herbal medicinal products since my childhood.			**.738**
% of variance explained	45.14	11.50	10.37

Extraction method: principle axe factor analysis. Rotation method: Promax with Kaiser normalisation. Rotation converged in seven iterations. Values below .1 not shown in the table. Bold numbers indicate the highest loadings per factor of the associated item

The reliability of the determined factors was tested by calculating Cronbach’s alpha values, which were acceptable and thereby provided evidence for internal consistency. Nunnally [39] recommended a 0.7 threshold, which has clearly been exceeded for each factor (pull factor .87; push factor .81; traditional factor: .80). The factors can therefore be used for further calculations. It is to be noted that deleting one item would not improve the Cronbach’s Alpha for each factor scale.

### Association between reason factors and new, established and long-term use of herbal medicine

Regarding the factors’ mean values in Table 4, the pull factor was found to be most agreed, as its value was the highest for all user groups, with means of 3.62, 3.93, and 4.10 for the group of new, established and long-term HM users respectively. The analysis of variance revealed statistically significant differences on the *p* ≤ .0005-level between the three user groups on two factors, namely the pull and the traditional one. Furthermore, results of a post-hoc comparison showed that the mean values of these two factors differed significantly among all groups. To be specific, for the pull and the traditional factors, the long-term user had a significantly higher level of agreement compared to the new and the established HM users. Moreover, the established user also had a significantly higher level of agreement for these two factors compared to the new user.

**Table 4:** Results of Welch’s ANOVA for the factors’ mean values

	User Group Means (SD)			
Factor	New user *(n = 166)*	Established user *(n = 1,482)*	Long-term user *(n = 544)*	Welch’s ANOVA (F ratio)
Pull factor	3.62 (0.83)	3.93 (0.70)	4.10 (0.72)	21.15 ****
Push factor	2.83 (0.89)	2.97 (0.87)	3.00 (0.91)	2.44
Traditional factor	2.70 (1.10)	3.07 (1.03)	4.00 (0.90)	245.32 ****

The Games-Howell post-hoc comparison showed that all groups differed significantly from each other for the pull and traditional factor. Mean = mean value; SD = standard deviation; **** *p* ≤ .0005

Results of the multinomial logistic regression analysis, comparing the influence of the three factors as independent variables on the dependent variable “user group”, are shown in Table 5. Concerning the overall model evaluation and goodness of fit statistics, the results show that the model is effective and fit the data for distinguishing between the groups: The likelihood ratio test yielded a χ^2^ value of 440.151 and a *p*-value of ≤.0005. Also, the Pearson measurement demonstrated statistical significance with a χ^2^ value of 2128.840 and a p-value of 0.001. Moreover, the overall correct classification rate was 70.4% and regarding Nagelkerkes R^2^, the model accounted for 22.7% of variance.

**Table 5:** Results of multinomial logistic regression analysis

	Established user (vs. new)			Long-term user (vs. new)			Long-term user (vs. established)		
Independent Variable	B	Adj. OR	95% CI	B	Adj. OR	95% CI	B	Adj. OR	95% CI
Push factor	−.210	.811	.637–1.032	−.478**	.620	.474–.811	−.268**	.765	.659–.888
Pull factor	.564**	1.758	1.335–2.316	.367*	.1.443	.1.044–1.995	−.197	.821	.669–1.008
Traditional factor	.255**	1.290	1.086–1.533	.1422**	4.144	.3.347–5.130	1.167**	3.211	2.786–3.702

B = regression coefficient b; Adj. OR = adjusted odds ratio. CI = confidence intervals for odds ratio. *p ≤ 0.05; **p ≤ 0.01. Pseudo R^2^: Cox und Snell: .182, Nagelkerke: .227, McFadden: .125

Compared to the new HM user, the established user is more likely to use HM due to reasons associated with pull or traditional aspects. Comparing the long-term user with the new user again indicates the importance of both pull and traditional aspects; in the same way, the long-term user is also more likely to use HM driven by pull or traditional aspects compared to the new HM user. Moreover, the results show that agreeing to items concerning the push factor reduces the likelihood of being a long-term vs. a new user. The comparison of the long-term user to the established one revealed that long-term users were less likely to take HM due to push aspects, but were more likely to use HM due to traditional aspects.

## Discussion

The strong increase in HM usage and its growing importance for medical care in recent years has underlined the need to better understand reasons underlying the decision making for initial and returning use of herbs in medicinal context. With more and more people using HM, it is interesting to know who is behind the newest “wave” of HM consumers. We found that these are mostly male, with a tendency of being less educated and having a medium/poor health-status. This fits the complementary finding of previous studies that people that have already decided on HM are predominantly female and well educated [40–42]: The latter implies that people not using HM are predominantly male and less educated, which means that people with these characteristics have a greater potential initiating HM usage, in agreement with our finding. It is mentioned that new users were more often unemployed, which could potentially impact their decision to use HM, since it is usually associated with smaller costs than many other CAM forms, such as chiropractic treatments. While our focus in this work was on the reasons for HM usage, further aspects of the user characteristics will be studied in future work.

Regarding the reasons for HM use, our results show a high level of agreement, with the top five agreed reasons all being related to the pull factor that is attracting consumers to HM because of its conceived positive aspects. Among these was the reason that HM is “healthier” than CM showing that patients have potentially underestimated the possible interaction and side effects of HM usage, which is an important safety issue that was often discussed in the literature. The least agreed reasons, on the other hand, are related to the push factor, which shows that the consumer decision for HM is not strongly impacted by conceived negative aspects of CM. Similarly, Sirois [43] found that there was a trend of an increasing importance of pull factors in guiding consumers’ choices for CAM, when comparing the agreement to reasons for deciding for CAM treatment between 1997/1998 and 2005.

Based on the results of the performed factor analysis, we found that three factors were underlying the 13 reasons we considered in our questionnaire: Factor 1 was related to reasons highlighting positive aspects of HM, factor 2 included reasons on negative aspects concerning CM, and factor 3 addressed reasons associated with traditional aspects, when using HM. Factor 1 and 2 are reminiscent of the “push” and “pull” factors which are well-known when analysing consumer behaviour in the context of CAM. However, the identification of factor 3, namely the importance of family traditions in HM use, is new. The importance of this factor for the decision making process of HM use in Germany can be understood by considering a longstanding use of herbs that is strongly embedded in the German culture.

Therefore, our study showed that the reasons towards the decision for HM use are more complex and that the simple push/pull model is not sufficient to explain it. While our study does not claim that the three factors will remain pertinent in all cases, it does highlight the importance of cultural context and family traditions in the consumer behaviour of HM users. In this context, it is interesting to note that previous studies showed that the family is an important and trustworthy source of information on HM [40, 44], showing that in the spirit of ref. [45], trust is a central aspect in the decision making for medical care.

Using logistic regression analysis, we found that compared to the new user, the long-term and established ones were positively associated with higher scores in the pull and traditional factor. In distinguishing the long-term from the established and new user, we found that higher push factor scores and higher traditional factor scores were negatively and positively associated with being a long-term user, respectively. Our results allow for the following summary concerning decision behaviour of HM users: the probability of being a new user compared to an established or long-term one is increased by higher scores on the push factors, i.e., a high level of dissatisfaction with CM has an important influence in prompting people to try HM. It is more relevant for new users than the positive aspects that are conceived with this type of medicine. However, positive experiences with HM support its users in maintaining their behaviour, and traditional aspects related to HM are an important factor for its long-term use. People who use HM because of traditional aspects that are important in their family environment simply do it for the reason that “it was always done this way”, which connects seamlessly to a long duration of HM usage. To what extent this framework can be related to previous findings in the literature is not a trivial question, since the picture of whether the push or the pull factors are more important overall or for initial/maintaining usage of CAM or HM is not entirely congruent. For example, Caspi et al. found that people would start CAM usage when CM treatment became ineffective [46]. Sirois et al. suggested that a high level of experienced dissatisfaction with CM, combined with intense symptoms, would prompt the decision for trying alternative treatment methods [14]. On the contrary, Bishop et al. [21] reported that the attraction to CAM is more influential than the dissatisfaction with CM.

Finally, we would like to address potential shortcomings of our study. First, we cannot exclude the possibility that the set of reasons we have examined is incomplete despite our careful survey design and data collection method. It should also be emphasized that our data has been collected in Germany and that our results are likely not completely transferable to other countries.

## Conclusion

A detailed investigation of the reasons for HM usage has been lacking up to now. With our exploratory approach and comprehensive analysis of the reasons that underlie the usage of HM, we aimed at providing a better conceptual basis that can guide future empirical studies related to the decision for this particular form of treatment. Our findings contribute to the ongoing discussion of whether the push or pull factors are more influential in the decision making process for this type of medicine. Remarkably, we found that a three factor structure going beyond the push and pull factors underlies the reasons explaining HM usage, with the third factor being related to family tradition in HM usage. Our results benefit medical practitioners as well as health-care providers, in particular due to the finding that new HM users are driven to use this form of treatment in part because of negative aspects they associate with using CM. In future studies, it will be interesting to examine the relevance of each of these three factors, as well as their potential interrelation, for HM use in the population of different countries with different cultural environments.

## Data Availability

The questionnaire and dataset used are available from the corresponding author upon reasonable request.

## Abbreviations

CAM: Complementary and alternative medicine
CM: Conventional medicine
HM: Herbal medicine
